# 

**Published:** 1892-02-06

**Authors:** 


					['ZJZnqrwich HOSPl.TAL
.asoow Western Inf/rua/iy
WiTH EXTRA HURSINB SUPPLEMENT.
No, 280. Vol. XI.] Saturday,
February 6th, 1892.
[One Penny.

				

